# Supplementary material for: Directed transport of neutrophil-derived extracellular vesicles enables platelet-mediated innate immune response
Source: Nat Commun. 2016 Nov 15;7:13464. doi: 10.1038/ncomms13464 (PMC5116072; doi:10.1038/ncomms13464)
Supplement: Supplementary Information — Supplementary Figures 1-8 [file ncomms13464-s1.pdf]

Supplementary Figure S1

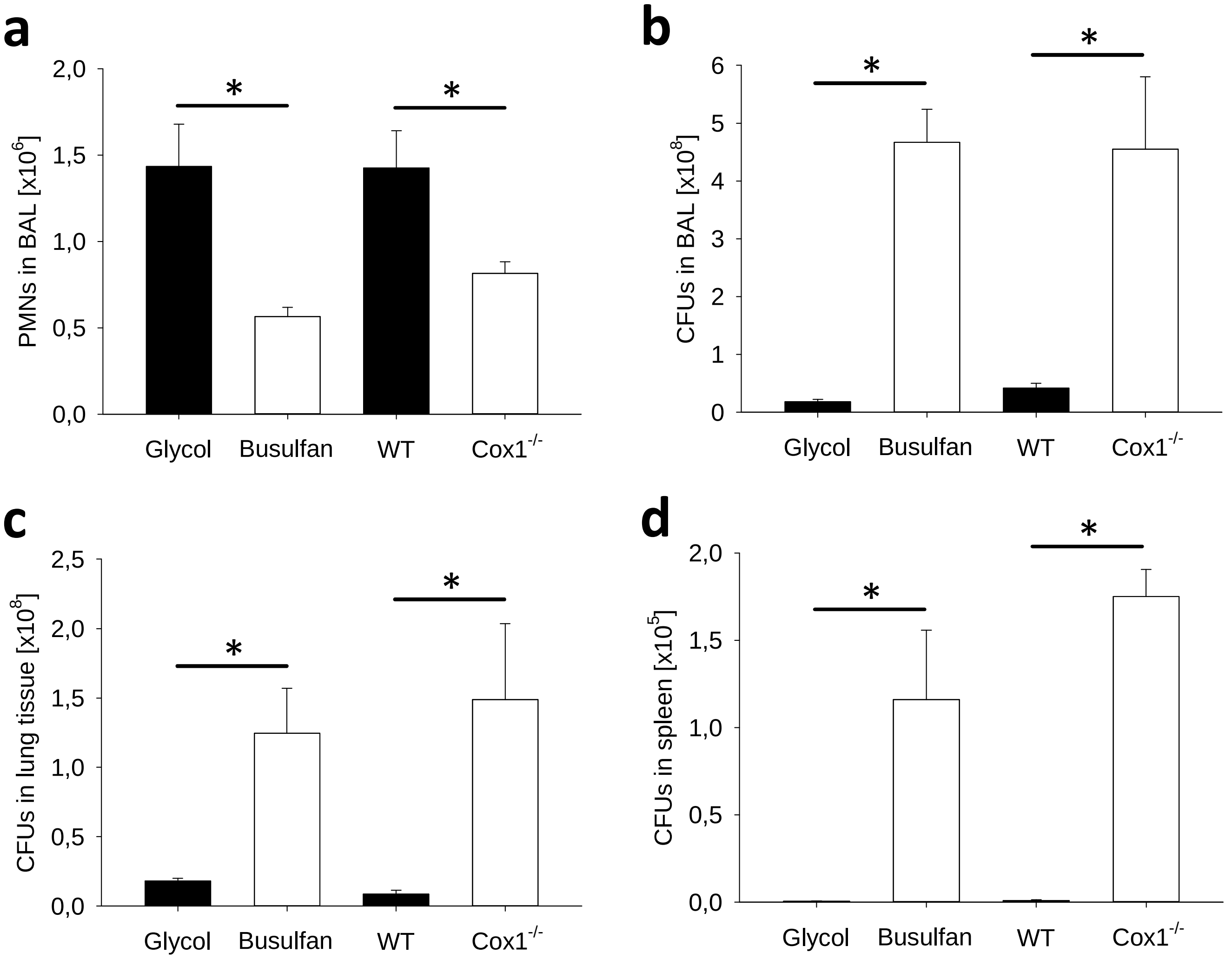

Supplementary Figure S1: **Platelets and Cox1 are required for neutrophil recruitment and pathogen control during pulmonary *K. pneumoniae* infections.** Glycol- and busulfan-treated wildtype as well as *Cox1*$^{-/-}$ mice were injected intratracheally with viable *K. pneumoniae*. **(a)** Neutrophil recruitment into the alveoli and the CFU count in the BAL **(b)**, lung tissue **(c)** and the spleen **(d)** were analyzed after 24 hours (n=4-5). Mean±SEM, ANOVA plus Bonferroni testing, * p <0.05

Supplementary Figure S2

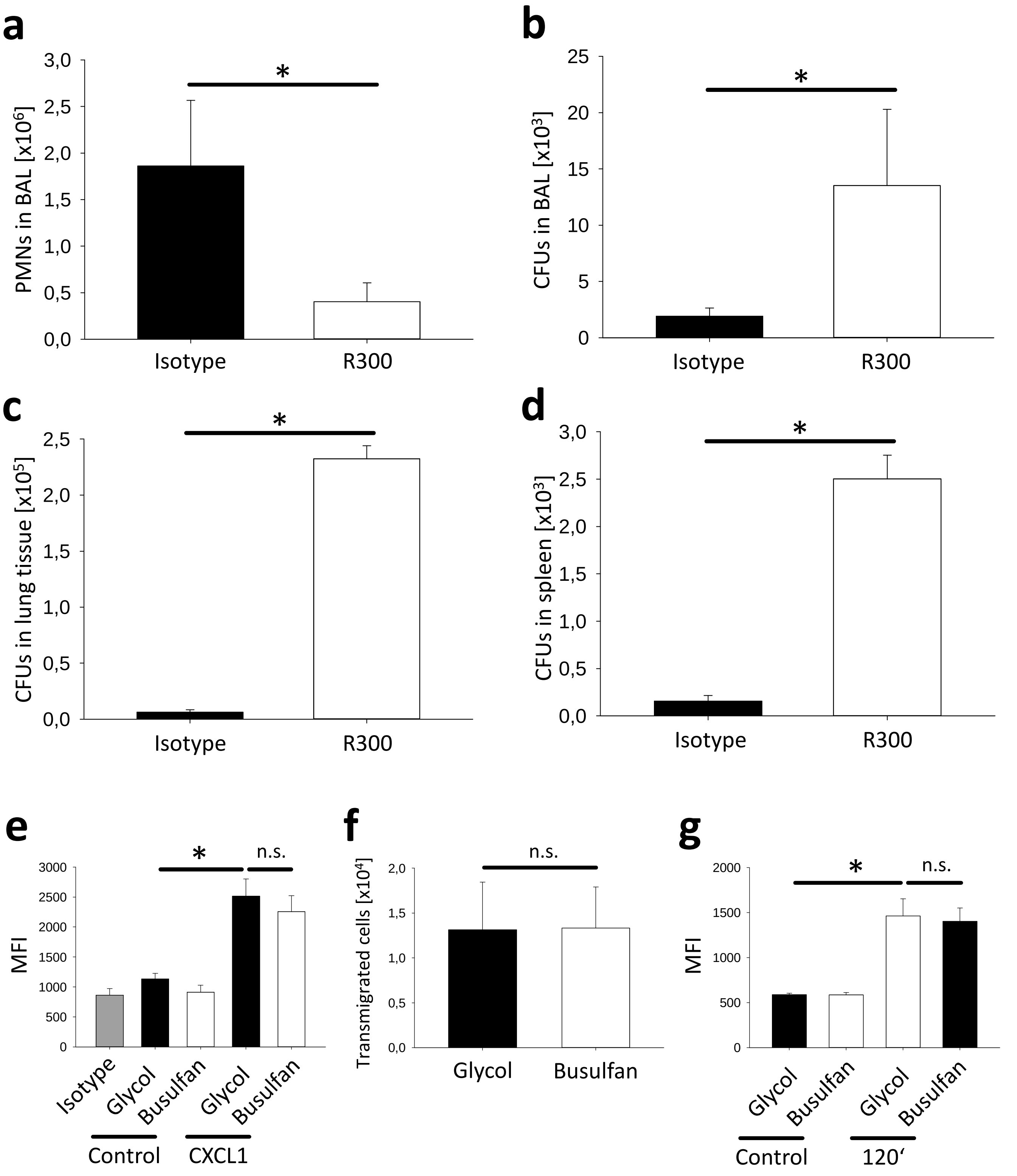

Supplementary Figure S2: **Busulfan treatment does not alter neutrophil function.** Isotype antibody- and platelet-depleting antibody-treated (R300) wildtype mice were injected intratracheally with viable *E. coli*. **(a)** Neutrophil recruitment into the alveoli and the CFU count in the BAL **(b)**, lung tissue **(c)** and the spleen **(d)** were analyzed after 24 hours (n=4-5). Neutrophils were isolated from glycol- or busulfan-treated mice and **(e)** ICAM1-binding (n=4), **(f)** transmigration (n=9) and **(g)** phagocytosis of pHrodo E. coli particles were analyzed (n=6). Mean±SEM, 2-tailed t test in S2a-d and S2f, ANOVA plus Bonferroni testing in S2e and S2g, * p <0.05

Supplementary Figure S3

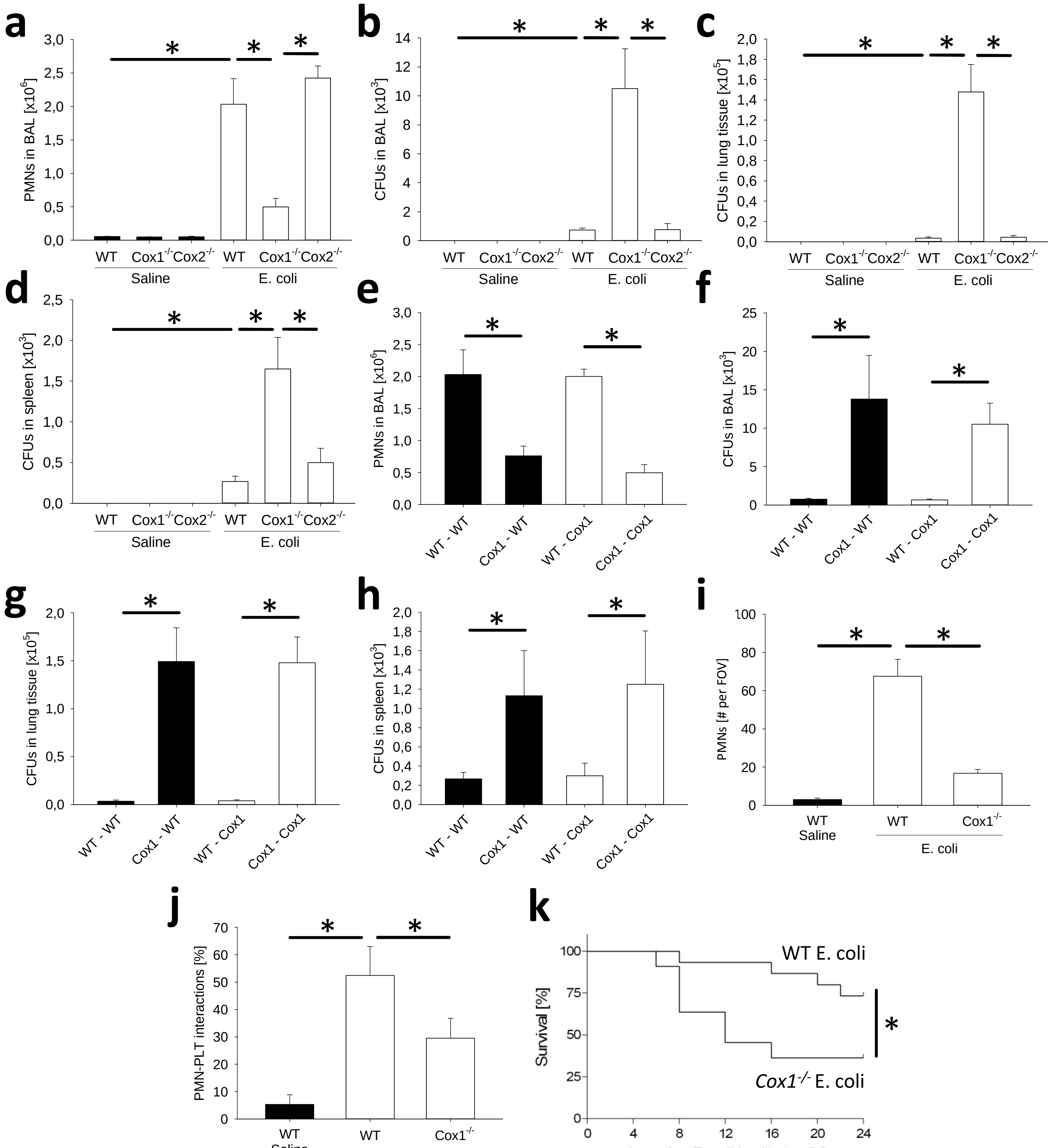

Supplementary Figure S3: **Hematopoietic Cox1 is indispensable to control bacterial spread.** WT mice, *Cox1*$^{-/-}$ and *Cox2*$^{-/-}$ mice were injected intratracheally with viable *E. coli*. **(a)** Neutrophil recruitment into the alveoli and the CFU count in the BAL **(b)**, lung tissue **(c)** and the spleen **(d)** were analyzed after 24 hours (n=4). Bone marrow chimeric mice (WT→WT, *Cox1*$^{-/-}$→WT, WT→*Cox1*$^{-/-}$, *Cox1*$^{-/-}$→*Cox1*$^{-/-}$) were generated by irradiation and bone marrow transplantation and **(e)** neutrophil recruitment into the alveoli and the CFU count in the BAL **(f)**, lung tissue **(g)** and the spleen **(h)** were analyzed after 24 hours (n=4). **(i)** Number of accumulated neutrophils per field of view in lung IVM (n=3). **(j)** Neutrophils interacting with platelets in the lung capillaries *in vivo* (n=3). **(k)** Survival 24 hours after instillation of $8x10^6$ viable *E. coli* (n=8-15). Mean±SEM, ANOVA plus Bonferroni testing in S3a-k, log rank test in S3k, * $p < 0.05$

Supplementary Figure S4

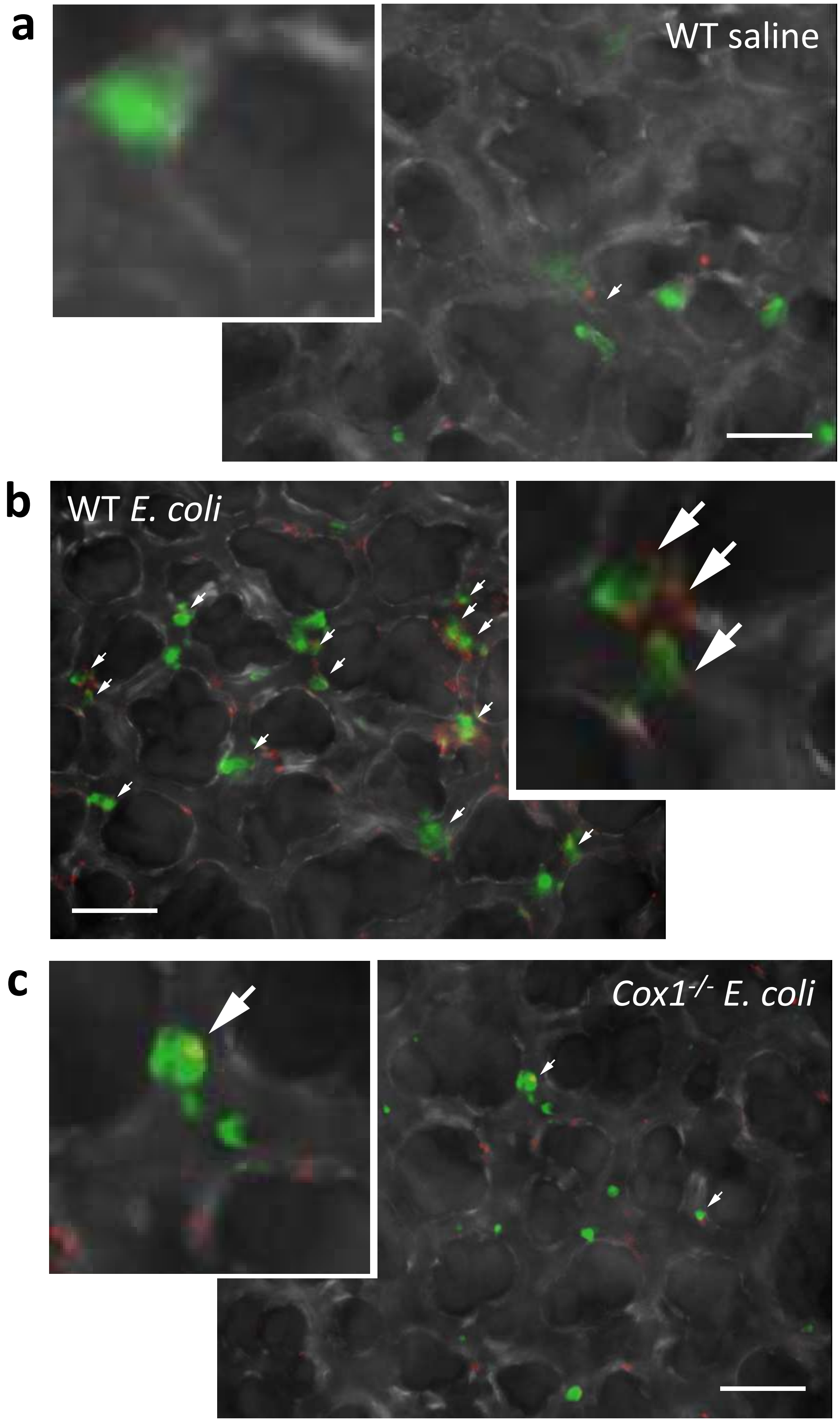

Supplementary Figure S4: **Lung intravital microscopy after *E. coli* instillation.** Mice were intratracheally instilled with *E. coli* or saline as a control. Neutrophils were labeled with Gr1-Alexa488 (green) and platelets were labeled with CD41-PE (red). Exemplary micrographs from WT mice treated with saline **(a)**, WT mice instilled with *E. coli* **(b)** and *Cox1*$^{-/-}$ mice instilled with *E. coli* **(c)**. Arrows indicate platelet-neutrophil interactions. Scale bar equals 50 µm.

Supplementary Figure S5

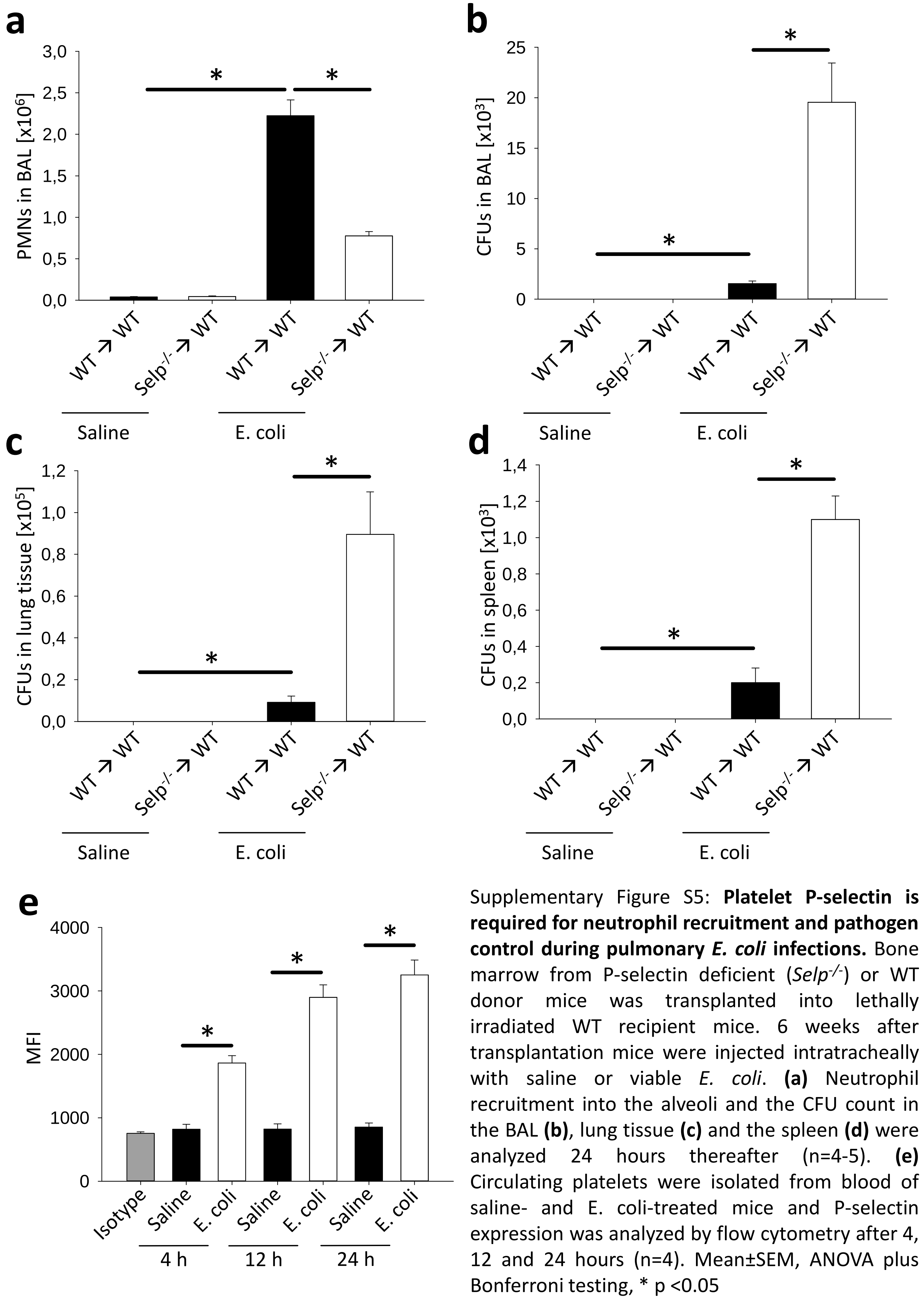

Supplementary Figure S5: **Platelet P-selectin is required for neutrophil recruitment and pathogen control during pulmonary *E. coli* infections.** Bone marrow from P-selectin deficient (*Selp*$^{-/-}$) or WT donor mice was transplanted into lethally irradiated WT recipient mice. 6 weeks after transplantation mice were injected intratracheally with saline or viable *E. coli*. **(a)** Neutrophil recruitment into the alveoli and the CFU count in the BAL **(b)**, lung tissue **(c)** and the spleen **(d)** were analyzed 24 hours thereafter (n=4-5). **(e)** Circulating platelets were isolated from blood of saline- and E. coli-treated mice and P-selectin expression was analyzed by flow cytometry after 4, 12 and 24 hours (n=4). Mean±SEM, ANOVA plus Bonferroni testing, * $p < 0.05$

Supplementary Figure S6

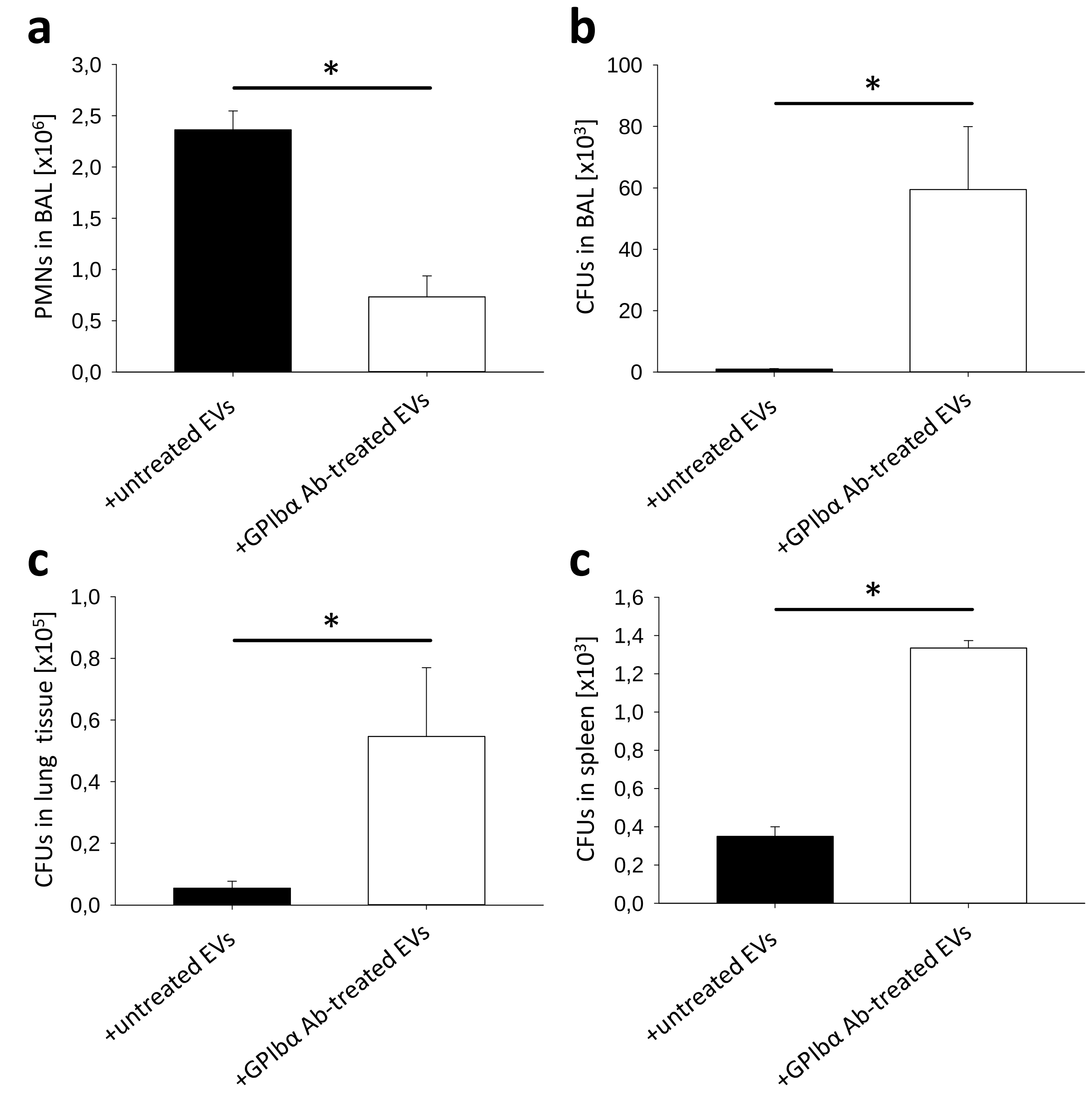

Supplementary Figure S6: **GPIbα on EVs is necessary for host defense.** Wildtype mice pretreated with the blocking antibody against GPIbα (clone Xia.B2, 50µg/mouse) were injected intratracheally with viable *E. coli* and reconstituted with either untreated isolated EVs or with isolated EVs that had been preincubated with the blocking antibody against GPIbα (clone Xia.B2) *in vitro*. Neutrophil recruitment into the alveoli **(a)** and the CFU count in the BAL **(b)**, lung tissue **(c)** and the spleen **(d)** were analyzed after 24 hours (n=4). Mean±SEM, 2-tailed t test, * $p < 0.05$

Supplementary Figure S7

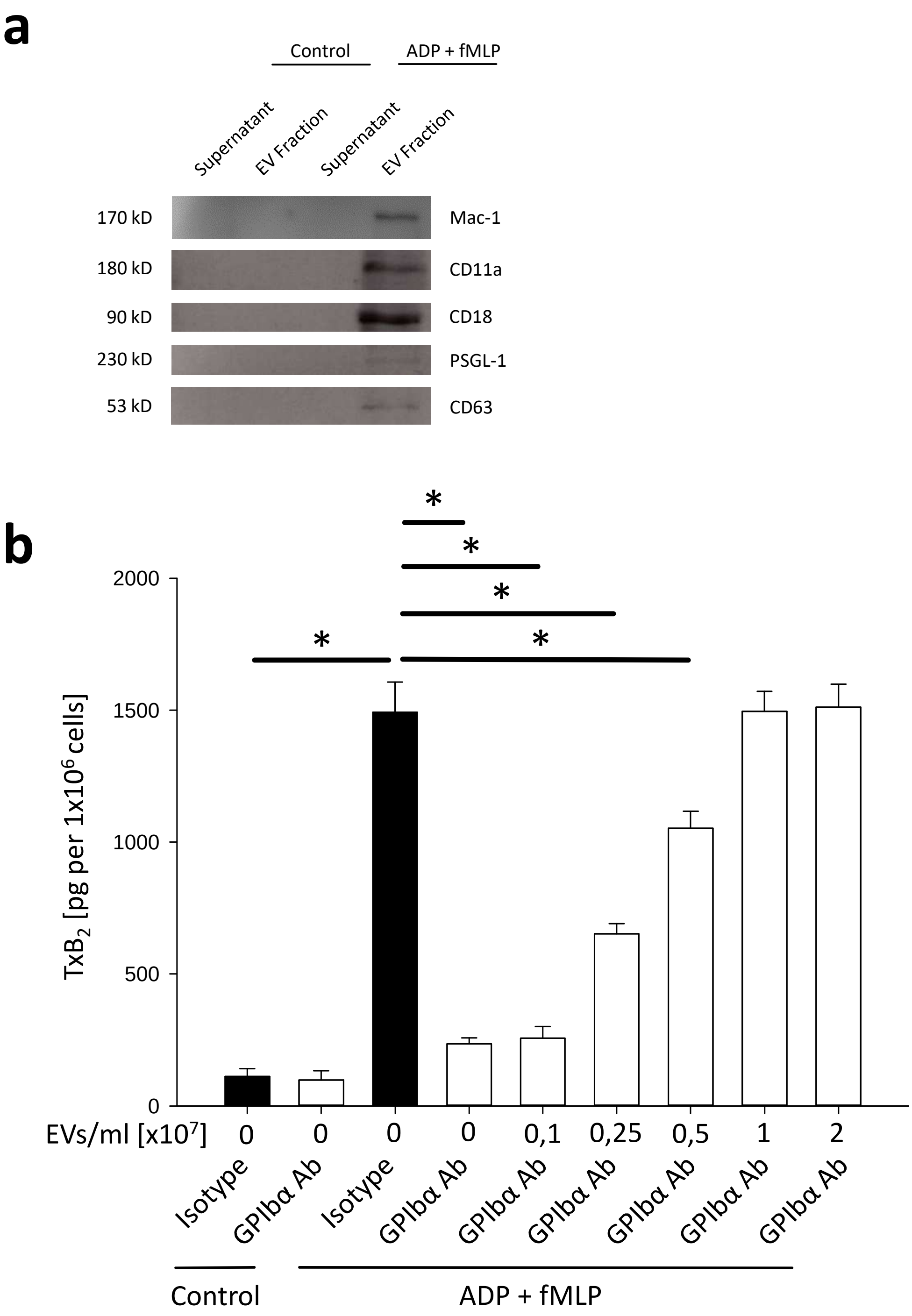

Supplementary Figure S7: **Characterization of neutrophil-derived EVs. (a)** Mac-1, CD11a, CD18, PSGL1, and CD63 in supernatant and EV fraction from control and stimulated samples was detected by western blot (exemplary blot from 3 experiments). **(b)** PMNs and platelets were isolated from WT mice and treated with a blocking antibody against GPIbα. Isolated neutrophil-derived EVs were reconstituted at the designated concentration and TxB_2 production in control and ADP/fMLP-stimulated samples was analyzed (n=3). Mean±SEM, ANOVA plus Bonferroni testing, * $p < 0.05$

Supplementary Figure S8

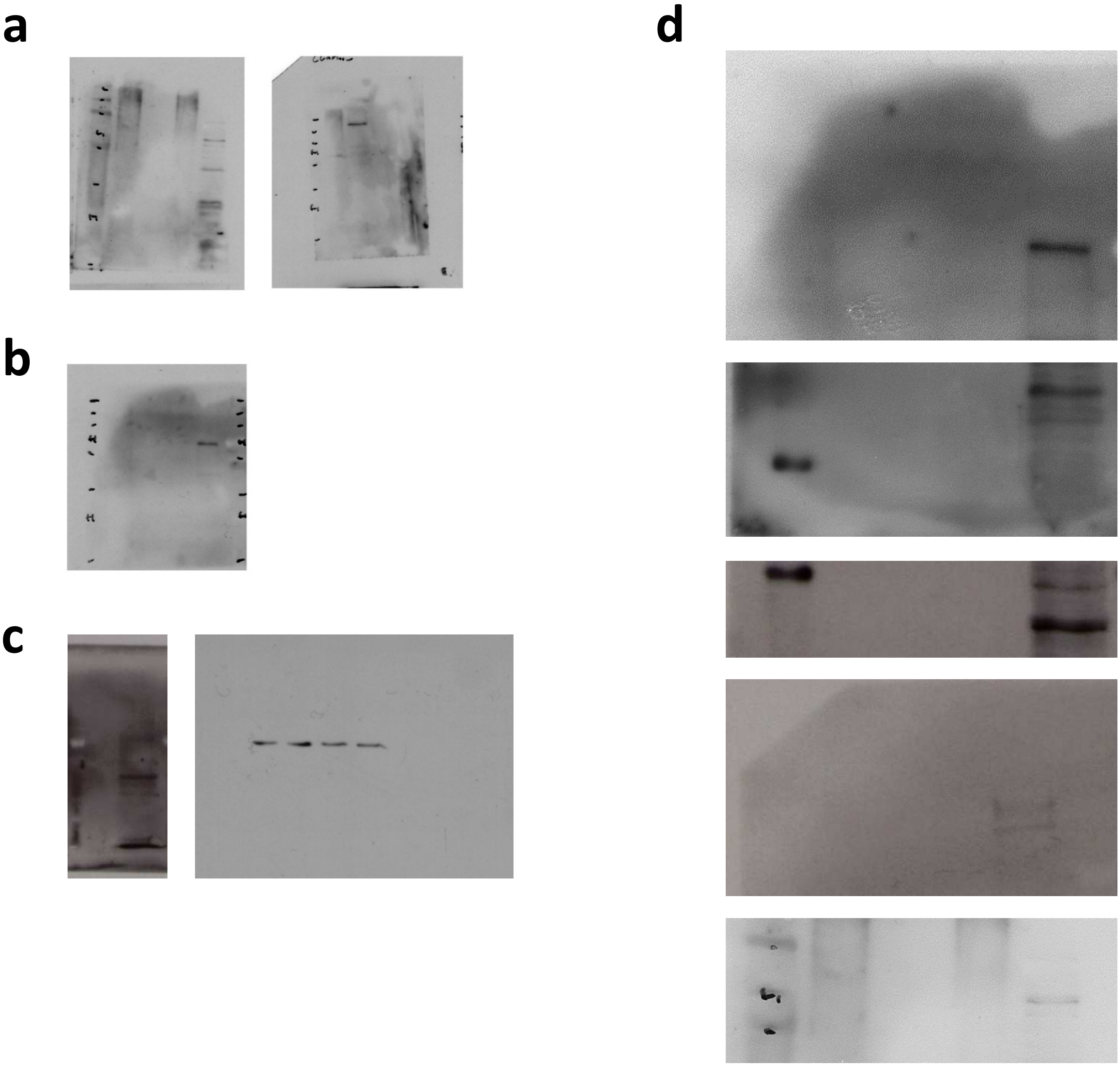

Supplementary Figure S8: **Uncut Western Blot gels. (a)** Blots corresponding to Figure 5a. Left blot: Caveolin-1. Right blot: Clathrin. **(b)** Blots corresponding to Figure 5d. **(c)** Blots corresponding to Figure 5g. Left blot: Mac-1. Right blot: Rap1. **(d)** Blots corresponding to Supplementary Figure S7. Blots in following order (from top to bottom): Mac-1, CD11a, CD18, PSGL-1, CD63.
